# Pain trajectory after total knee arthroplasty (TKA) with prolonged use of outpatient adductor canal catheters

**DOI:** 10.1016/j.jatmed.2025.07.001

**Published:** 2025-08-01

**Authors:** Shane Barre, Anwar Alinani, Jose Chu Luo, Waiman Liu, Bethany Fink, Haylee Herbaugh, Sanjib Adhikary

**Affiliations:** a Department of Anesthesiology and Perioperative Medicine, Penn State Health Milton S. Hershey Medical Center, 500 University Dr., Hershey, PA 17033. USA; b Australian National University, Research School of Finance, Actuarial Studies & Statistics, Canberra, ACT 2600, Australia

**Keywords:** Adductor canal catheter, Orthopedic anesthesia, Acute pain, Regional anesthesia, Total knee arthroplasty

## Abstract

**Background:**

In this prospective observational study, we sought to evaluate whether the prolonged use of adductor canal nerve block catheters provides benefits for patients undergoing primary total knee arthroplasty.

**Methods:**

Patients aged 18 or older with an ASA score of 1–3 undergoing single primary total knee replacement were deemed eligible. After TKA procedure, adductor canal catheters were placed in the post-anesthesia care unit. Patients were followed for five consecutive days postoperatively. Inpatient data were obtained from the electronic medical record. After discharge, data were collected from daily phone calls. The main parameters were the average numeric pain score (0–10) at rest, the average numeric pain score (0−10) during and after physical therapy, and daily opioid consumption.

**Results:**

A total of 797 patients consented to take part in the study, 336 were lost to follow up, and 461 patients were included in the study. Among those with adductor canal catheters in place the average pain scores both at rest and during physical therapy decreased on Day 2 and Day 3. The average pain levels during physical therapy were significantly lower (p < 0.05) in patients with the catheter in place compared to those without, and opioid consumption was also decreased on these days.

**Conclusions:**

This study demonstrates that the prolonged use of adductor canal nerve block catheters may reduce pain and opioid consumption in patients undergoing primary TKA for up to 72 h postoperatively.

## Introduction

Each year approximately 800,000 total knee replacements are performed in the United States. Projections estimate a 673 % increase by 2030, reaching approximately 3.5 million procedures annually.^1^, ^2^ At present, the trend is to discharge most of these patients either on the same day or within 24 h of surgery. Pain management is a vital component to prepare patients ready for discharge. The current trend in acute pain management is to tailor protocols according to anticipated postoperative pain trajectory.

The postoperative pain trajectory after surgery depends on multiple factors, including patient characteristics, surgical approach, and pain management strategies utilized. For example, while intense pain following total hip arthroplasty typically resolves quickly, pain after total knee arthroplasty (TKA) often follows a more protracted course with moderate and severe pain persisting for weeks.^3^, ^4^, ^5^, ^6^ Recently, several different analgesic techniques for TKA have evolved. These include, but are not restricted to, non-opioid and opioid medications along with local anesthesia infiltration by surgeons as a part of multimodal analgesia techniques. As epidurals, femoral nerve blocks, popliteal (sciatic) nerve blocks, and fascia iliaca blocks have fallen out of favor for patients undergoing TKA, primarily due to motor blockade, regional anesthetic techniques such as adductor canal nerve blocks, infiltration between popliteal artery and capsule of the knee (IPACK), and genicular nerve blocks have become more popular largely due to their motor-sparing effects.^7^ However, the selection of these techniques varies significantly among institutions.

Adductor canal nerve blocks have been proven to be an effective pain management strategy after total knee arthroplasty.^8^, ^9^, ^10^, ^11^, ^12^ Most studies evaluating continuous adductor canal nerve block catheters maintain infusions for only 24–48 h.^13^, ^14^ Evidence also suggests that use of a continuous adductor canal block catheter is superior to single injection blocks.^15^ Given the prevalence of prolonged pain following TKA, we investigated whether extended use of continuous catheters provides clinical benefits.

In this prospective study, we followed up patients who received adductor canal nerve blocks for TKA in our institution for up to five days post operatively. To our knowledge, this is the first study to follow patients with adductor canal catheters for this length of time. The primary goal of this study was to determine the effectiveness of these nerve blocks by following the patient’s daily numeric pain scores at rest, during and after physical therapy as well as their daily opioid consumption in morphine milligram equivalents (MME). We also sought to evaluate the typical duration of these nerve block infusions after leaving the hospital.

## Materials and methods

This IRB approved prospective study was completed at Penn State Health Milton S. Hershey Medical Center between October 2019 to July 2022. STROBE guidelines were used to ensure clear presentation and organization of this study.^16^ Informed verbal consent was obtained by all participating patients on postoperative day 1. This occurred in person if the patient was still in the hospital or by phone call if the patient had been discharged. Eligible participants included English-speaking patients aged > 18 years scheduled for primary total knee replacement. Those undergoing revision, unicompartmental, or bilateral total knee replacements were excluded. After discussion with the patients, anesthesia type (spinal vs. general) was determined by the attending anesthesiologist taking care of the patient. Following our institution’s standard protocol, patients provided consent for postoperative adductor canal nerve block catheter placement in our post anesthesia care unit (PACU). Surgeons administered the Total Knee Arthroplasty, “TKA solution,” at doses of their own discretion during the procedures. TKA solution consisted of ropivacaine 0.5 % (50 mL), ketorolac 30 mg (1 mL), epinephrine 1 mg/mL (0.1 mL), and NaCl 0.9 % 48.9 mL. Adductor canal catheters were placed with ultrasound guidance mostly by anesthesia residents under the direct supervision of an attending anesthesiologist. Bolus and infusion dosing of the adductor nerve block catheters was at the discretion of the anesthesia attending supervising the block. All nerve block infusions consisted of 0.2 % ropivacaine, and all patients received either continuous infusions with patient-controlled boluses or autoboluses with patient-controlled boluses. Patients were followed for five consecutive postoperative days. While patients remained inpatient, all data was able to be extracted from our electronic medical record. Once discharged from the hospital, post-operative data were collected from daily phone calls conducted by a member of the research team. The following data were collected daily: the average numeric pain score (0–10) at rest, the average numeric pain score (0−10) during and after physical therapy, all non-opioid and opioid analgesics taken each day, and the number of times physical therapy or prescribed exercise were performed. The date and time of nerve block infusion completion and nerve block catheter removal were also recorded.

Data analyses and management were executed in STATA 18© statistical software (StataCorp LP, College Station, TX, USA). We used the ordinary least square regression technique to estimate differences in pain scores and amount of morphine dosage between the two groups after adjusting for age, BMI, gender and ASA physical status. Since we tested pain level and morphine use over multiple time points, there is an increased rate of false positives. To control for false discovery, we applied Benjamini-Hochberg procedure (Benjamini and Hochberg (1995)) to adjust for a false discovery rate of 5 %. Benjamini-Hochberg procedure is preferred over classic approach such as Bonferroni, as the latter tends to over-penalize multiple tests, leading to higher rate of false negative. A coefficient is considered significant if its p-value is less than the Benjamini-Hochberg critical value.^16^

## Results

**Fig. 1:**
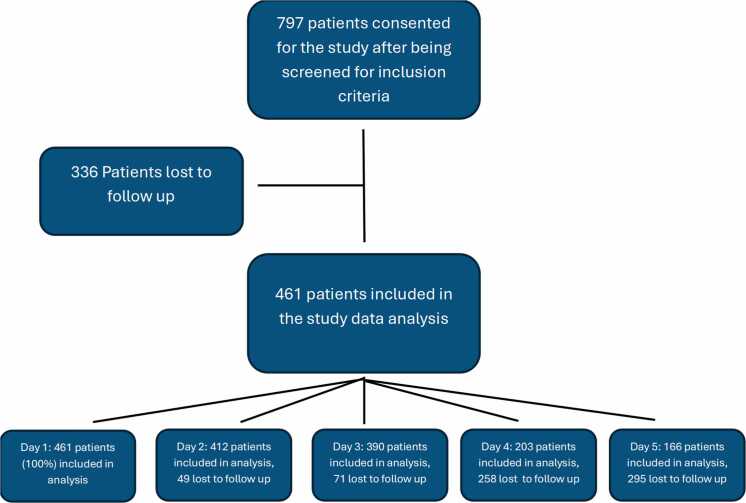
Patient flow diagram.

**Fig. 2:**
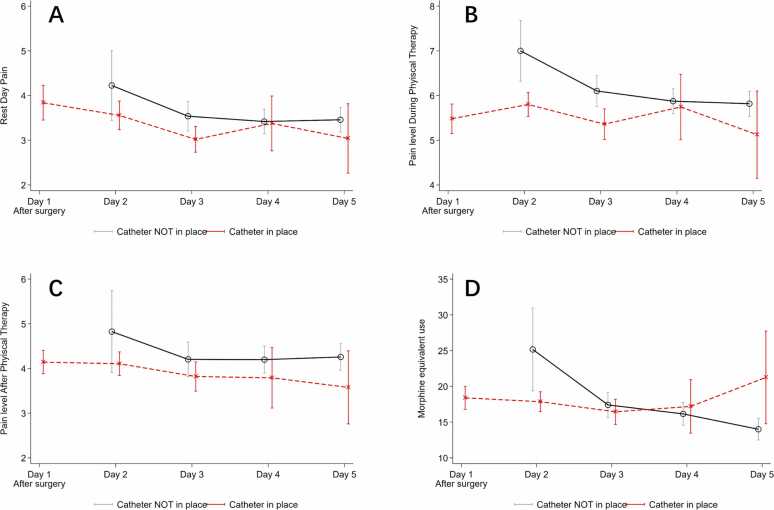
Pain level. (A) Pain level at rest. (B) Pain level during physical therapy. (C) Pain level after physical therapy. (D) Morphine equivalent use.

As shown in Fig. 1, a total of 797 patients consented to take part in the study after being pre-screened against the inclusion criteria using electronic health records. A total of 336 patients were lost to follow up due to failure to answer post-operative phone calls, resulting in data analysis being performed on 461 patients. Table 1 presents the demographics of the enrolled participants, including age, gender, BMI, ASA score, and type of anesthetic used during the procedure. Fig. 2 Panels A–C presents the average pain level at rest, during and after physical therapy, and their corresponding 95 % confidence interval over time. Panel D presents the average morphine equivalent usage in milligrams (mg).

**Table 1:** Demographics.

**Variable**	**Mean**	**SD**
Age	65.9	9.2
BMI	32.9	5.8
	Number (N)	Percentage (%)
Gender		
Female	[258]	[56]
Male	[203]	[44]
ASA		
1	[3]	[1]
2	[239]	[52]
3	[218]	[47]
Anesthetics		
GA	[179]	[39]
SP	[259]	[57]
Both SP and GA	[20]	[4]

GA: General Anesthesia, SP: Spinal Anesthesia.

Table 2 presents the average pain level and morphine equivalent usage (in mg) from POD 1 to POD 5, and the corresponding *p*-values on comparing the groups with and without after adjusting for patient demographics, i.e. age, BMI, female gender, and ASA status. On the first day, all 461 study participants still had their nerve block catheter in place. On POD 2, 49 study participants (11 %) were lost to follow-up because they did not answer phone calls on the day after the operation, leaving a total of 412 data points. Of 412 patients, 90 % (*n* = 370) of them still had their nerve block catheters in place, and the percentage fell to 55 % (*n* = 215) on POD 3, 29 % (*n* = 58) on POD 4 and 16 % (*n* = 26) on POD 5.

**Table 2:** Home-catheter program, pain level and morphine use.

**Variables**	**Catheter in place**				
	**Yes**	**No**	**Diff (Yes – No)**	**Adjusted Diff**^**§**^	***p*****-value**^**§**^
**Day 1**					
*N*	461	0			
Pain level - At rest	3.84	-	-	-	-
- PT	5.48	-	-	-	-
- Post PT	4.14	-	-	-	-
Morphine equivalent use	18.39	-	-	-	-
**Day 2**					
*N*	370	42			
Pain level - At rest	3.56	4.22	−0.67	−0.60	0.21
- PT	5.80	7.00	−1.20	−1.09	< 0.01*
- Post PT	4.11	4.82	−0.72	−0.60	0.22
Morphine equivalent use	17.85	25.16	−7.31	−7.08	< 0.01*
**Day 3**					
*N*	215	175			
Pain level - At rest	3.02	3.54	−0.52	−0.50	0.12
- PT	5.36	6.10	−0.74	−0.65	**0.04**
- Post PT	3.82	4.20	−0.38	−0.32	0.11
Morphine equivalent use	16.45	17.41	−0.96	−1.08	0.02*
**Day 4**					
*N*	58	145			
Pain level - At rest	3.38	3.42	−0.04	0.03	0.90
- PT	5.75	5.87	−0.13	−0.09	0.68
- Post PT	3.79	4.20	−0.41	−0.31	< 0.01*
Morphine equivalent use	17.19	16.15	1.04	1.08	0.70
**Day 5**					
*N*	26	140			
Pain level - At rest	3.04	3.46	−0.42	−0.33	< 0.01*
- PT	5.13	5.82	−0.69	−0.50	< 0.01*
- Post PT	3.58	4.26	−0.68	−0.50	0.05
Morphine equivalent use	21.25	14.00	7.25	6.99	0.01*

The differences and their corresponding p-values are estimated using ordinary least square after adjusting for age, BMI, Female gender and ASA. Statistically significant (*) based on the Benjamini Hochberg cutoffs due to false discovery rate. The total missing catheter data on Day 3, 4 and 5 are 8, 23 and 21, respectively.

On POD 1, the average numeric pain score (0–10) at rest, during physical therapy and after physical therapy were 3.84, 5.48 and 4.14, respectively. A few notable patterns emerged from the table. Among those with catheter in place, the average pain scores, both at rest and during physical therapy, decreased on Day 2 and Day 3, and the average pain levels during physical therapy were significantly lower (*p* < 0.05) in patients with the catheter in place. The use of catheters reduced their pain level and lowered the requirement of opioid usage (p < 0.05) on those days. On Day 4, there were no statistical differences between the two groups in terms of pain levels (both at rest and during physical therapy) and morphine equivalent consumption; however, there was a statistically significant difference in pain scores after physical therapy. On Day 5, the pain level before and during physical therapy was significantly lower in those who still had their catheter in place and yet, their morphine consumption was significantly higher.

## Discussion

The results of this study demonstrate that it may be beneficial to keep an adductor canal catheter in place for up to three days postoperatively.

To our knowledge, this is the first study to evaluate pain control up to five days postoperatively in patients undergoing primary TKA surgery with in-situ adductor canal catheters. Our study also highlights the successful implementation of a home regional catheter program for adductor canal catheters after TKA. This is despite the current apparent skepticism regarding discharging patients with prolonged lower extremity blockade.^17^ We documented a 100 % success rate of the adductor canal catheters still being in place on POD 1. However, we presume this is due to the fact that all our catheters were placed in the PACU after surgery, and patients were not very ambulatory except for their required physical therapy sessions. It is interesting to see that a majority of patients were even able to keep their catheters in situ for 72 h. There were a small number of patients who kept their catheters up to the 4th and 5th postoperative day. This was likely due to different infusion strategies and patient-controlled dosing options. Patients who used patient-controlled dosing often used up their infusions faster and removed their nerve block catheters sooner.

One of the most noteworthy findings of our study was that most patients had significant pain after primary TKA for at least 72 h, and often beyond. Therefore, our study shows that the prolonged use of continuous adductor canal catheters may have a stronger role in managing effective pain control over these patients while reducing their opioid requirements. The previous metanalysis by Hussain et al. suggested that continuous catheter-based adductor canal block does not enhance or prolong the analgesic benefits when compared with single-shot adductor canal block for TKA over the first 48 h postoperatively. ^18^ Even though our study did not compare continuous adductor canal blocks to single shot blocks, it shows that more prolonged use of adductor canal catheters may be warranted due to the protracted pain trajectory after TKA.

Studies evaluating pain trajectory after hip surgery have demonstrated that after 6–8 h, severe pain tends to subside.^4^ This is in contrast with TKA where severe pain may last 72 h or longer. This is a particularly important finding, as the current surgical trend is to discharge patients on the same day of surgery or within 24 h. This finding will help to construct a procedure-specific pain curve to guide clinicians on the timing of advanced analgesic measures in patients undergoing TKA. Similarly, it would likely be beneficial to study pain trajectory in all types of surgery so that analgesia can be optimized and tailored to the specific type of surgery.

This study has a number of limitations. First, it is an observational study, hence none of the interventions were randomized. We did, however, adjust for patient characteristics including age, BMI, gender, and ASA score as discussed in the methods section. All patients received 0.2 % ropivacaine for their infusions; however, their infusion strategies varied, and they could also receive patient-controlled bolus options. Differences between infusion strategies were not evaluated in this study. In addition, adjunct medication use by each patient was not standardized. The amount of TKA solution used by each surgeon also varied. After patient discharge, data collection relied on phone calls to the patients, which had its own limitations. We chose this method in the hope of ensuring more compliance and reliability compared to other methods that would require patients to self-report their data. Many patients did not routinely pick up their phones, and this could have been for various reasons. Most phone calls were placed in the evening, though it was impossible to know when patients would be available. In the future, data could be collected more objectively with wearable and/or digital monitoring devices, which could help obtain more robust and reliable data. Patient factors also likely played a significant role in collecting data. It is possible that patients with worse pain control may have been more likely to answer postoperative phone calls, whereas those with better pain control may not have wanted to be bothered, however, this is merely speculation.

## Conclusions

This study affirms the fact that TKA can leave patients with significant pain for many days after surgery despite multimodal analgesic strategies. Adductor canal nerve blocks have been shown to be efficacious for patients undergoing TKA; however, the use of adductor canal catheters versus single shot blocks remains a matter of debate. In addition, the optimal duration of adductor catheter use and dosing strategies for either catheters or single shots has not been determined. Our study shows that adductor canal catheters can be effectively left in place for up to five days postoperatively on an outpatient basis. Our data suggests there may be a benefit to the prolonged use of adductor canal catheters for up to 3 postoperative days in patients undergoing TKA. Further randomized and controlled studies are needed to confirm the optimal duration and dosing strategies of postoperative adductor canal nerve block catheters after TKA.

## CRediT authorship contribution statement

**Shane Barre:** Writing – review & editing, Writing – original draft, Resources, Project administration, Methodology, Investigation, Data curation, Conceptualization. **Anwar Alinani:** Visualization, Project administration, Methodology, Investigation, Formal analysis, Data curation, Conceptualization. **Jose Chu Luo:** Writing – review & editing, Project administration, Investigation, Data curation. **Waiman Liu:** Writing – review & editing, Formal analysis, Data curation. **Bethany Fink:** Writing – review & editing, Project administration, Data curation. **Haylee Herbaugh:** Writing – review & editing, Project administration, Data curation. **Sanjib Adhikary:** Writing – review & editing, Visualization, Supervision, Project administration, Methodology, Investigation, Conceptualization. All authors have read and agreed to the published version of the manuscript.

## Consent for publication

Informed verbal consent was obtained by all participating patients on postoperative day 1.

## Ethical statement

The study was approved and deemed exempt by the Institutional Review Board (or Ethics Committee) of Penn State Health Milton S. Hershey Medical Center and Penn State College of Medicine (protocol code 00014365 date of approval: 7 February 2020).

## Funding

This research received no external funding.

## Declaration of competing interest

The authors declare that they have no known competing financial interests or personal relationships that could have appeared to influence the work reported in this paper.

## Data Availability

The data that support the findings of this study are available from the corresponding author upon reasonable request.
